# Using disability-adjusted life years to set health-based targets: A novel use of an established burden of disease metric

**DOI:** 10.1057/jphp.2013.22

**Published:** 2013-05-30

**Authors:** Katherine Gibney, Martha Sinclair, Joanne O'Toole, Karin Leder

**Affiliations:** a Department of Epidemiology and Preventive Medicine, The Alfred Centre, 99 Commercial Road, Melbourne VIC3004, Australia.

**Keywords:** burden of illness, health-based targets, health policy, disability-adjusted life years, Australia, drinking water

## Abstract

Following the 1990 Global Burden of Disease (GBD) study, Disability-Adjusted Life Years (DALYs) have been used widely to quantify the population health burden of diseases and to prioritise and evaluate the impact of specific public health interventions. In the context of the recent release of the 2010 GBD study, we explore the novel use of DALYS to determine health-based targets (HBTs). As with the more traditional use of DALYs, the main advantage of using DALYs as HBTs is the ability to account for differential disease severity, identify the most appropriate public health interventions, and measure the positive and negative outcomes of these interventions. Australia is currently considering adopting DALYs for setting HBTs for drinking water quality, as recommended by the WHO. Adoption of DALY HBTs could be relevant in other areas, including air quality, food safety, health care-associated infections, and surgical complications.

Following the 1990 Global Burden of Disease (GBD) study,^1^ Disability-Adjusted Life Years (DALYs) have been widely used to quantify the population health burden of diseases and to prioritise and evaluate the impact of specific public health interventions. In the context of the recent release of the 2010 GBD study,^2^ we explore the novel use of DALYS to determine health-based targets (HBTs).

DALYs measure the population impact of specific health conditions, accounting for both premature death and morbidity. One DALY equates to 1 lost year of ‘healthy' life. The DALY metric quantifies the gap between a population's current health status and an ideal where everyone lives to advanced age, free from disease and disability.^3^ DALYs are the sum of years of life lost (YLL) due to premature death and years lost due to disability (YLD) for incident cases of the particular health condition in a population (*DALY=YLL+YLD*). Calculation of YLL requires information on the number of people who died from the disease and their life expectancy at age of death. YLD incorporates the number of incident cases, symptom duration, and symptom severity (the ‘disability weight' that ranges from 0 for perfect health to 1 for death). Differing health outcomes for a single health condition (that is, differing severity levels and disease sequelae) can be incorporated into the DALY model.

The DALY metric has been used for quantification and comparison of the burden of diseases in a population; comparison of disease burdens between countries, regions, and population groups; comparison of the impact of risk factors (for example, smoking and obesity) on disease burden; and prioritisation and evaluation of public health interventions.^4^,^5^

When DALYs are used to set HBTs, it is necessary to nominate a tolerable population DALY burden, or acceptable risk; calculate the average burden of a single case of disease (the DALY/case); and determine the tolerable number of disease cases (see Figure 1). As with the population-level DALY metric, calculation of the average DALY/case allows consideration of various disease outcomes. Waterborne gastrointestinal pathogens, for example, are chosen as reference pathogens when setting microbial HBTs for water quality.^6^ Acute gastroenteritis has four possible courses – mild, moderate, or severe disease, and death. In addition, gastroenteritis caused by certain pathogens (for example, *Campylobacter*) can be followed by sequelae such as Guillain-Barré syndrome, reactive arthritis, and irritable bowel syndrome.^7^ The contribution of these sequelae to the disease-specific DALY/case can exceed that of the acute gastroenteritis.^8^ By considering the frequency and duration of different possible disease outcomes and the relevant disability weights, the average DALY/case can be calculated for each reference pathogen. Different pathogens have different average DALY/case impacts because of their unique morbidity and mortality characteristics.

The WHO Guidelines for Drinking Water Quality (GDWQ) define safe drinking water as ‘not representing any significant risk to health over a lifetime of consumption'.^6^,^9^ They promote the use of DALYs to determine HBTs, stating that provision of safe drinking water should involve: (i) setting HBTs; (ii) ensuring adequate and properly managed systems; and (iii) providing independent surveillance. The tolerable disease burden set in the GDWQ is 10^−6^ DALYs per person-year, meaning that each reference pathogen in drinking water should not cause the loss of more than 365 healthy days in a population of one million people in a year.^6^ Reference pathogens are chosen to represent viruses, bacteria, and protozoa, based on criteria that include: being sufficiently well characterised in terms of dose-response, infectivity, and disease outcomes; occurrence in source waters and sensitivity to removal or inactivation by treatment processes; and having a high public health impact.^6^,^10^ To determine the level of water treatment required to meet this HBT, DALY/case values for each reference pathogen and the tolerable number of water-related disease cases caused by that pathogen must first be defined. Quantitative microbial risk assessment (QMRA) is then applied to determine the likelihood of infection or illness following exposure to the reference pathogen in drinking water. Finally, pathogen surveillance data for source waters is combined with QMRA and DALY/case data to quantify the amount of source water treatment required to achieve adequate pathogen reduction to meet the HBT.

Although the WHO GDWQ first published in 2004 the recommendation for use of DALYs in setting HBTs for drinking water quality,^9^ no country has adopted this approach in national drinking water or other health-related guidelines, other than the Australian Guidelines for Water Recycling (AGWR).^11^ The current Australian Drinking Water Guidelines provide quantitative (non-DALY) health-based guideline values for chemical and radiological water contaminants, but lack quantitative targets for microbial water quality.^12^ In Australia, therefore, quantitative microbial HBTs are currently defined if highly treated sewage effluent, stormwater, or greywater is used to supplement drinking water supplies (covered in the AGWR), but not for drinking water drawn from conventional water sources.

An alternative HBT used in water safety guidelines by the US Environmental Protection Agency, Health Canada, the Netherlands, and New Zealand is the *annual infection risk* approach, with an accepted risk of one infection per 10 000 person-years. This target relates only to infection, not to the occurrence, severity, or outcome of symptomatic disease. Similar to the DALY/case approach, the annual infection risk approach identifies reference waterborne gastrointestinal pathogens, uses QMRA to determine the likelihood of infection following exposure, and applies surveillance data concentrations to quantify the required pathogen reduction.

When used for setting microbial HBTs, the DALY/case approach takes into consideration the potential public health impact of each pathogen, whereas the annual infection approach treats all pathogens as equally significant. In a study examining the disease burden attributable to foodborne pathogens in the Netherlands in 2009, the highest number of disease cases were attributed to norovirus, rotavirus, *Staphylococcus aureus*, and *Clostridium perfringens*; whereas the greatest population disease burden (DALYs) were attributed to *Toxoplasma gondii*, *Campylobacter*, rotavirus, norovirus, and *Salmonella*.^8^ Differences were due to differential disease severity, age at death for fatal cases, and disease burden from sequelae related to these pathogens, resulted in *Campylobacter* causing a higher average disease burden per case (41 DALYs per 1000 cases) than rotavirus (4·9) or norovirus (2·4). Differences in impact-ranking of pathogens are even more pronounced if number of infections (rather than number of symptomatic cases) is compared with DALYs. The prevalence of asymptomatic norovirus infection has been reported at 12 per cent among healthy individuals in England.^13^ Therefore, if norovirus were used as a reference pathogen to set HBTs using either an annual infection or DALY/case approach, more water treatment resources would be consumed to reduce the norovirus load to meet the annual infection target (because of high numbers of asymptomatic and relatively mild cases) compared with the DALY/case approach (relatively low DALY/case for norovirus). When used to set water-related HBTs, the DALY/case approach might reduce requirements for unnecessary and costly water treatments.^6^ An additional advantage of the DALY/case approach is that it can compare potential public health impacts posed by disparate health risks (for example, microbial, chemical, or radiological contamination of water supplies), along with health impacts and economic costs of proposed interventions.^6^,^14^ Furthermore, the application of DALYs as HBTs is not limited to water guidelines; other potential areas include air quality, food safety, health care-associated infections, and surgical complications. Use of DALY HBTs for non-microbial health conditions, such as those associated with exposure to radiation or chemicals, is currently limited due to knowledge gaps.^6^

There are limitations to using DALYs in setting HBTs. First, this was not the intended use of the DALY metric, as it was developed to quantify the population burden of disease and injury.^15^ Second, HBTs are often somewhat arbitrary and require a degree of value-judgement, which may be country- or situation-specific (for example, there is lack of consensus on the target of 10^−6^ DALYs per person-year included in the WHO GDWQ).^16^ Similarly, disability weights used in different studies vary and revised disability weights from the 2010 GBD study were recently released.^2^,^17^ Inherent uncertainties in DALY estimates are due to limited data on number, duration, and potential for sequelae following disease cases of each severity.^8^ In addition, there are uncertainties in QMRA models and insufficient data regarding pathogen concentrations in source waters (necessary elements for implementation of DALY HBTs).^6^,^10^ Use of HBTs requires selection of reference conditions and extrapolation of DALY/case estimates to other related conditions: for water guidelines, water treatment requirements for an entire class of pathogens (for example, viruses, bacteria, or protozoa) are based on DALY/case calculations for a single reference pathogen within each class, relying on the premise that water treatment options that control the reference pathogens are expected to control other important pathogens within each pathogen class.^10^ Uncertainties regarding use of DALY HBTs also apply to other HBT approaches such as the annual infection approach, including the judgement-based nature of determining tolerable risk, QMRA uncertainty, and the use of reference pathogens to represent an entire pathogen class.

In conclusion, while DALYs were developed and have been widely used to compare diseases within and between populations and to prioritise and evaluate public health interventions, this metric can also be used to set meaningful HBTs. As with the more traditional use of DALYs, the main advantage of using DALYs as HBTs is the ability to account for differential disease severity and to prioritise and measure public health interventions more meaningfully. Australia is currently considering adopting DALYs for setting HBTs for drinking water quality,^18^ as has been recommended by the WHO.^6^ Adoption of DALY HBTs could also be relevant in other areas.

## Figures and Tables

**Figure 1:**
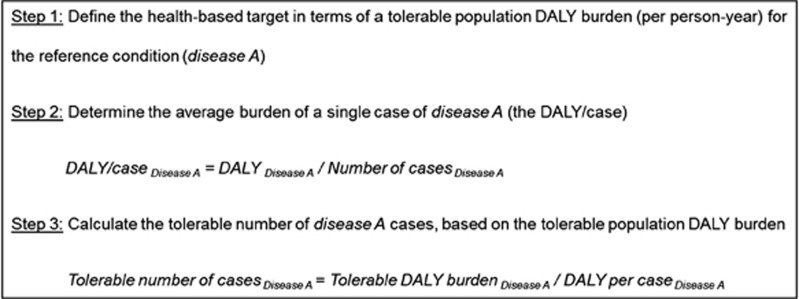
Use of DALYs to set health-based targets.
